# Independent risk factors for myasthenic crisis and disease exacerbation in a retrospective cohort of myasthenia gravis patients

**DOI:** 10.1186/s12974-022-02448-4

**Published:** 2022-04-12

**Authors:** Christopher Nelke, Frauke Stascheit, Carmen Eckert, Marc Pawlitzki, Christina B. Schroeter, Niklas Huntemann, Philipp Mergenthaler, Ercan Arat, Menekse Öztürk, Dirk Foell, Stefanie Schreiber, Stefan Vielhaber, Asmae Gassa, Henning Stetefeld, Michael Schroeter, Benjamin Berger, Andreas Totzeck, Tim Hagenacker, Sven G. Meuth, Andreas Meisel, Heinz Wiendl, Tobias Ruck

**Affiliations:** 1 Department of Neurology, Medical Faculty, Heinrich Heine University Düsseldorf, Moorenstraße 5, 40225 Duesseldorf, Germany; 2 Charité, Universitätsmedizin Berlin, corporate member of Freie Universität Berlin, Department of Neurology With Experimental Neurology, Humboldt-Universität zu Berlin, Berlin, Germany; 3 Charité, Universitätsmedizin Berlin, corporate member of Freie Universität Berlin, NeuroCure Clinical Research Center, Humboldt-Universität zu Berlin, Berlin, Germany; 4 Department of Neurology with Institute of Translational Neurology, University and University Hospital Münster, Munster, Germany; 5 Department of Child and Adolescent Psychiatry and Psychotherapy, University Hospital Münster, Munster, Germany; 6 Department for Pediatric Rheumatology and Immunology, University of Münster, Munster, Germany; 7 Department of Neurology, University of Magdeburg, Magdeburg, Germany; 8 Department of Cardiothoracic Surgery, University of Cologne and University Hospital Cologne, Cologne, Germany; 9 Department of Neurology, University of Cologne, Faculty of Medicine and University Hospital Cologne, Cologne, Germany; 10 Clinic of Neurology and Neurophysiology, Medical Center, Faculty of Medicine, University of Freiburg, Freiburg, Germany; 11 Department of Neurology and Center for Translational Neuro- and Behavioral Sciences (C-TNBS), University Hospital Essen, University of Duisburg-Essen, Essen, Germany; 12 Charité, Universitätsmedizin Berlin, corporate member of Freie Universität Berlin, Center for Stroke Research Berlin, Humboldt-Universität zu Berlin, Berlin, Germany; 13 German Myasthenia Gravis Society, Berlin, Germany; 14 German Center for Neurodegenerative Diseases, Bonn, Germany; 15 Center for Behavioral Brain Sciences, Magdeburg, Germany

**Keywords:** Myasthenic crisis, Myasthenia gravis, Disease exacerbation, Risk factors, Predictors

## Abstract

**Background:**

Myasthenic crisis (MC) and disease exacerbation in myasthenia gravis (MG) are associated with significant lethality and continue to impose a high disease burden on affected patients. Therefore, we sought to determine potential predictors for MC and exacerbation as well as to identify factors affecting outcome.

**Methods:**

We examined a retrospective, observational cohort study of patients diagnosed with MG between 2000 and 2021 with a mean follow-up of 62.6 months after diagnosis from eight tertiary hospitals in Germany. A multivariate Cox regression model with follow-up duration as the time variable was used to determine independent risk factors for MC and disease exacerbation.

**Results:**

815 patients diagnosed with MG according to national guidelines were included. Disease severity at diagnosis (quantitative MG score or Myasthenia Gravis Foundation of America class), the presence of thymoma and anti-muscle specific tyrosine kinase-antibodies were independent predictors of MC or disease exacerbation. Patients with minimal manifestation status 12 months after diagnosis had a lower risk of MC and disease exacerbation than those without. The timespan between diagnosis and the start of immunosuppressive therapy did not affect risk. Patients with a worse outcome of MC were older, had higher MGFA class before MC and at admission, and had lower vital capacity before and at admission. The number of comorbidities, requirement for intubation, prolonged mechanical ventilation, and MC triggered by infection were associated with worse outcome. No differences between outcomes were observed comparing treatments with IVIG (intravenous immunoglobulin) vs. plasma exchange vs. IVIG together with plasma exchange.

**Conclusions:**

MC and disease exacerbations inflict a substantial burden of disease on MG patients. Disease severity at diagnosis and antibody status predicted the occurrence of MC and disease exacerbation. Intensified monitoring with emphasis on the prevention of infectious complications could be of value to prevent uncontrolled disease in MG patients.

**Graphical Abstract:**

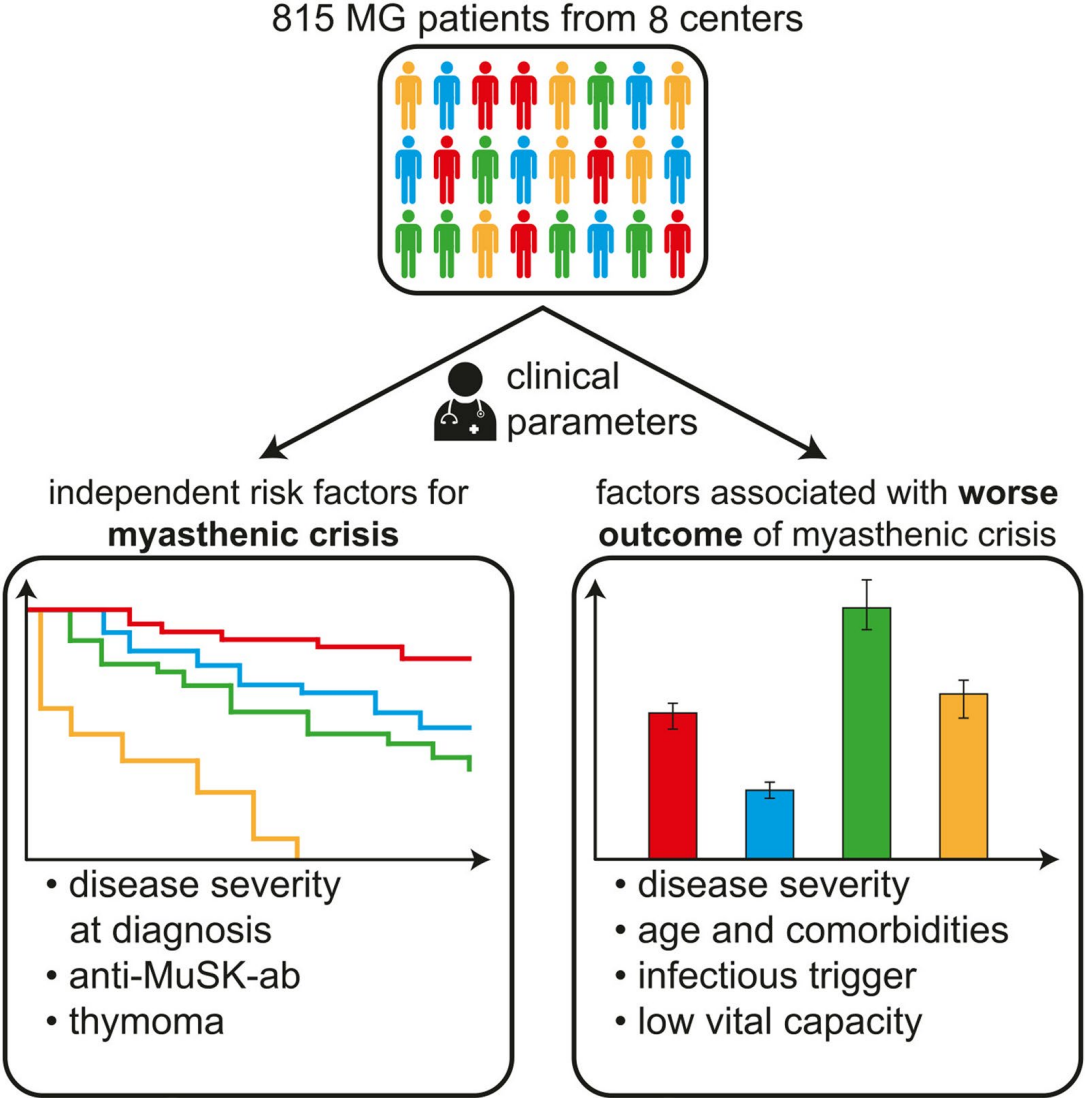

**Supplementary Information:**

The online version contains supplementary material available at 10.1186/s12974-022-02448-4.

## Background

Myasthenia gravis (MG) is an acquired autoimmune disorder of the neuromuscular junction characterized by dysfunction of the post-synaptic membrane [1]. Owing to improved treatment strategies and diagnostic tools, therapeutic outcomes have improved for the majority of MG patients [2]. However, a clinically distinct subgroup of patients, often referred to as refractory, remains symptomatic despite therapy [2, 3]. Exacerbation of disease and myasthenic crisis (MC) are frequent in these patients and substantially contribute to disease burden [4]. Despite diagnostic and therapeutic advances for the management of MG, patients experiencing MC continue to face a substantial mortality rate of approximately 5–12% [5, 6]. The requirement for hospitalisation, the associated burden of disease and the cost of available rescue therapies, underline the importance of the prevention and management of MC [7, 8].

Hindered by the rarity of MG, our understanding of the underlying pathophysiological mechanisms related to insufficient disease control remains fragmented. A range of potential triggers for the manifestation of MC or disease exacerbations have been observed including infections, surgery, adverse effects of medication, co-morbidity, pregnancy or tapering of immunosuppressive medication [9, 10]. Prognostic factors identifying patients at risk for MC or disease exacerbations remain incompletely understood and have only been characterized for MG patients presenting with a thymoma [11, 12]. However, factors predicting the occurrence of MC especially in patients without thymoma remain largely elusive. Finally, factors defining the outcome of MC are incompletely identified, but urgently needed to guide the clinical management of these patients. Our analysis aims at understanding factors predicting clinical deteriorations. We therefore analysed a cohort of 815 MG patients to identify potential risk factors for MC and disease exacerbations.

## Methods

### Study design and participants

**Fig. 1:**
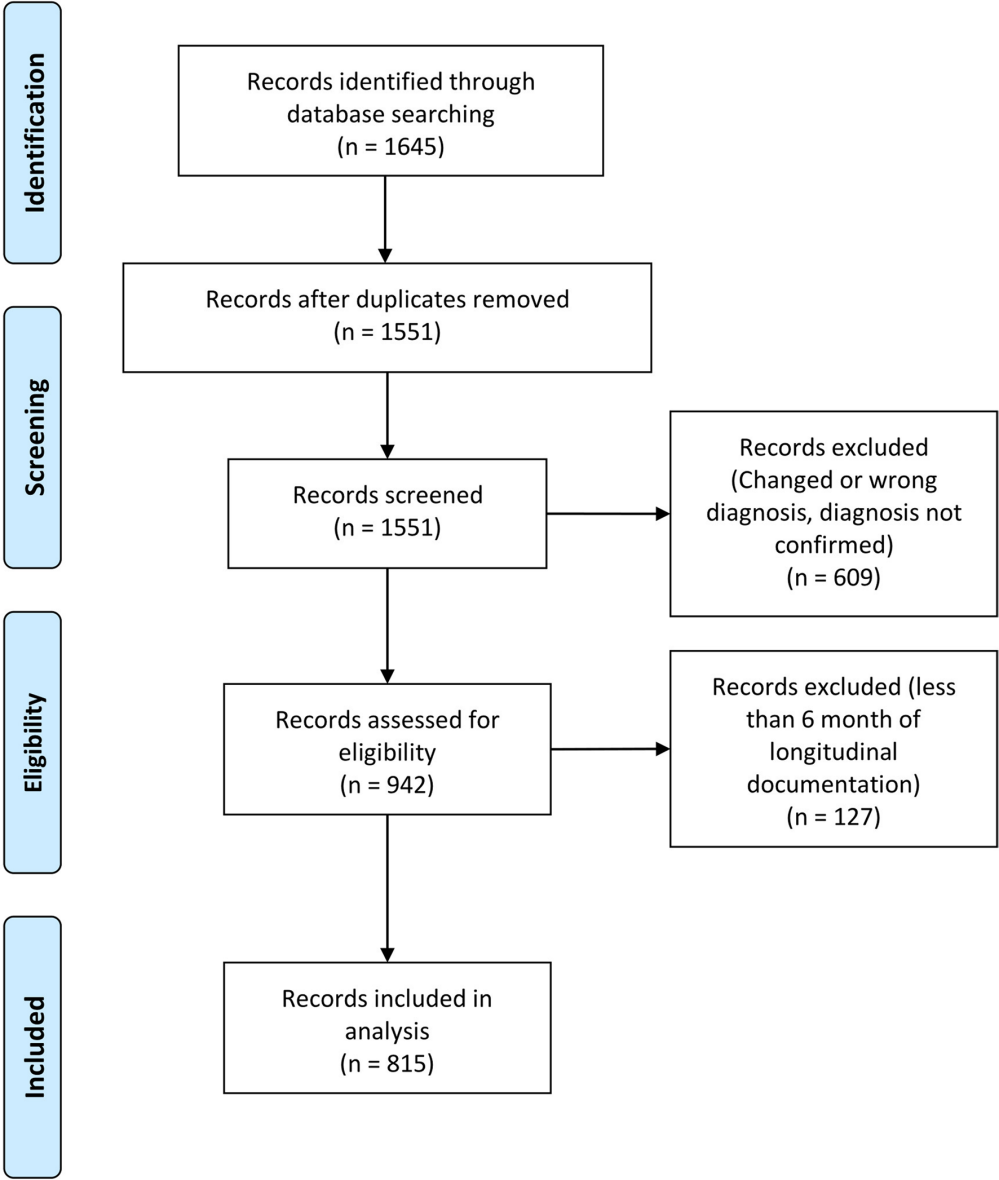
PRISMA flow chart detailing screening and inclusion of patient records for this study

Our cohort study is a retrospective analysis of 815 patients from eight university hospitals in Germany (Charité—Universitätsmedizin Berlin and University Hospitals Cologne, Duesseldorf, Essen, Freiburg, Magdeburg, Muenster and Regensburg). Patients requiring intensive care were treated on specialized neurological intensive care units (NICU). Patients were identified by searching the on-site database for the corresponding ICD-10 code (ICD-10-GM-2019 G70.-). Overall, 1645 patients were screened, of whom 815 were included in the analysis (Fig. 1). Diagnosis of MG was established by characteristic clinical presentation in accordance with national guidelines [13], independent of disease duration or severity. All centres are certified as integrated myasthenia centre (iMC) by the German Myasthenia Gravis Society applying standardised clinical pathways for patient management. Diagnosis was supported by antibody findings and repetitive nerve stimulation. Antibody testing was performed by enzyme-linked- or radio-immunoassay (Euroline). Suspected cases without established diagnosis, with a change to their diagnosis (*n* = 609) or with insufficient case documentation were excluded (< 6 months of longitudinal documentation) (*n* = 127) (Fig. 1). The final cohort consisted of patients diagnosed between January 2000 and July 2021. Patients with an established diagnosis and sufficient longitudinal documentation of > 6 months were included during this time period. Socio-demographics (age, sex, disease duration), antibody (ab) status (acetylcholine-receptor (AChR), muscle specific receptor tyrosine kinase (MuSK), lipoprotein-related protein 4 (LRP4), seronegative), MG specific medication (cholinesterase-inhibitors, glucocorticoids, and long-term immunosuppressant’s), history of thymoma-status, and comorbidities were collected from patient’ charts. The follow-up strategy was standardized across centres. According to iMC standards, patients with a stable course were seen every 6 months and instable patients more frequently. MG-specific scoring was performed by the treating neurologist at the time of presentation.

### Definitions

For this cohort analysis, we differentiated between MC and disease exacerbation as distinct clinical events.

A MC was defined as a rapid clinical decline requiring non-invasive ventilation, intubation or parenteral nutrition [14]. Dysphagia severe enough to require a nasogastric tube was also included as criterion for MC.

A disease exacerbation was defined as fulfilment all of the following criteria as adapted from national guidelines [15]:

- Objective: QMG (quantitative myasthenia gravis) score [16] of ≥ 8 points and a minimum increase of ≥ 5 points from the previous visit. Ocular findings must not account for more than 5 points on the QMG score.
- Subjective: progressive clinical deterioration due to weakness of bulbo-pharyngeal or limb muscles or reduced respiratory function impacting activities of daily living.
- Period of time: progress of symptoms no longer than 30 days.

A clinical event matching both the definition of MC and disease exacerbation was classified as MC. The outcome of MC was defined according to the MGFA (Myasthenia Gravis Foundation of America) post-intervention status (MGFA–PIS) [17, 18]: Specifically, improved signifies that QMG score at hospital discharge was reduced by ≥ 3 points compared to pre-admission, worse signifies that QMG score hospital discharge was increased by ≥ 3 points compared to before the admission and unchanged signifies that neither the criteria for improved nor worse were met. Patients with worse outcome were discharged for further rehabilitation. The threshold was defined to be that a score of 3 points in a single item of the QMG score reflects severe impairment [18]. The cutoff between early-(EOMG) and late-onset (LOMG) MG was set at 50 years as previously defined [19]. Minimal manifestation status (MMS) was defined in accordance with the MGFA–PIS as no symptoms of functional limitation from MG but weakness on examination only detectable by examination [17, 20, 21]. For MMS, immunosuppressive therapy and symptomatic therapy, e.g., cholinesterase inhibitors, were permitted (analogous to MMS-3 as proposed by the MGFA–PIS) [17, 20, 21].

### Standard protocol approvals, registrations, and patient consents

The study was approved by the local ethics committee and institutional review boards (no. AZ 2020-010-f-S, no. AZ 07/2017, 19-8973-BO, AZ 21-1265, AZ 21-1331). Data were anonymized and collected retrospectively according to the standardized requirements of the German register for myasthenia.

### Statistical analysis

Statistical Analysis was performed using GraphPad Prism 9.3 (GraphPad Software, Inc., San Diego, CA) and R (R Core Team, 2020). Data were presented as median (IQR = interquartile range), mean (standard deviatio*n* = SD), or *n* (%). For univariate logistic regression, goodness of fit was assessed by Cox-Snell’s generalized R squared or Tjur’s Pseudo R squared as appropriate. Significance was assessed by the likelihood ratio test. The odds ratio (OR) was assessed using a multivariate Cox regression model with follow-up as the time variable. Experiencing at least one MC or disease exacerbation compared to no event was used as the status variable. For analysis of time between diagnosis and MC or disease exacerbation the Kaplan–Meier method was used. Statistical significance between survival curves was determined by a pairwise log rank test. Analysis of variance (ANOVA) testing was performed for the analysis of groups for continuous variables and Fisher’s exact test for categorial variables. To account for multiple comparisons, statistical significance was corrected by the false discovery rate (FDR). Anonymized data will be shared by request from any qualified investigator. For regression analysis of MGFA class II to IV, MGFA classes A and B were combined to allow for statistical analysis. Therefore, analysis is limited to MGFA classes without distinguishing the distribution of muscle weakness.

## Results

### Baseline characteristics and clinical features

Clinical and demographic data are presented in Table 1. Mean age at disease onset was 52.7 years (SD 20.0) and at diagnosis 53.5 years (SD 19.8). Early disease onset before the age of 50 years occurred in 300 patients (36.8%), while 510 cases (62.6%) were LOMG. The follow-up time was 62.6 months (SD 73.3) after diagnosis.

**Table 1:** Clinical and demographic baseline characteristics of patients

Characteristic	*n*	%
Total	**815**	**100**
Sex		
Male/Female	361/454	44.4/55.6
Age, y		
Mean age at first manifestation, years	52.7 ± 20.0	
Mean age at diagnosis, years	53.5 ± 19.8	
Early-onset MG (< 50 years)	300	36.9
Decremental response		
Positive	368	45.2
Negative	446	54.8
Increment		
Positive	6	0.7
Generalized MG at diagnosis		
Ocular MG	215	26.3
Generalized MG	589	72.7
MGFA class at diagnosis		
I (ocular)	236	28.9
II	309	37.9
III	169	20.8
IV	43	5.3
V	25	3.0
Missing	30	3.7
QMG-score at diagnosis, median ± IQR	4.0 (2.0–8.0)	
Antibody status		
Seronegative	86	10.5
Seropositive	714	87.6
Anti-AchR-ab	641	89.9
Anti-MuSK-ab	71	9.9
Anti-LRP4-ab	2	0.3
Anti-Titin-ab	156	21.8
Missing	15	1.8
Thymectomy	294	36.1
MRI or CT		
Thymoma-suspect	98	12.0
Histology		
Thymoma	158	19.4
First IST		
Azathioprine	475	58.2
MMF	46	5.6
Methotrexate	41	5.0
Cyclosporine	0	0
Mean corticosteroid dosage following diagnosis, mg	15 ± 10	
Concomitant diseases		
Cardiovascular	379	46.6
Arterial hypertension	289	35.4
Heart failure (any cause)	59	7.3
Aortic stenosis	64	7.8
Cardiac arrythmia	45	5.5
Other	111	13.6
Pulmonary	133	16.3
Chronic obstructive pulmonary disease	74	9.1
Asthma	28	3.5
Smoking	89	10.9
Other	23	2.8
Metabolic	185	22.7
Diabetes mellitus (type 1 or 2)	166	20.4
Hypercholesterolemia	155	19.0
Other	21	2.5
Gastrointestinal	167	20.5
Celiac disease	18	2.2
Gastroesophageal reflux disease	91	11.1
Liver failure (any cause)	15	18.4
Inflammatory bowel disease	8	0.9
Other	12	14.7
Malignancy other than thymoma	91	11.2
Lung cancer	22	2.7
Prostate cancer	33	4.0
Breast cancer	12	1.4
Other	15	1.8
Autoimmune disease	152	18.7
Hashimoto's disease	44	5.3
Rheumatoid arthritis	32	3.9
Psoriasis	34	4.2
Multiple sclerosis	3	0.3
Other	22	2.7
Months of follow-up	62.6 ± 73.3	

Baseline characteristics of included patients with myasthenic syndromes. *ab* antibody, *anti-AChR-ab* anti-acetylcholine-receptor-ab, *anti-MuSK-ab* anti-muscle-specific tyrosine kinase-ab, *anti-LRP4-ab* anti-low-density lipoprotein receptor-related protein 4-ab, *MC* myasthenic crisis, *MG* myasthenia gravis, *MMF* mycophenolate-mofetil, *IST* immunosuppressive therapy, *IQR* interquartile range, *SD* standard deviation. Unless otherwise reported, values are mean ± SD (range), median ± IQR (range) or *n* (%); QMG-score = quantitative myasthenia gravis-score

MGFA class at diagnosis was available for 782 patients (96.3%). 236 (28.9%) patients presented with ocular weakness (Class I); 309 (37.9%) with mild symptoms (Class II); 169 (20.8%) with moderate symptoms (Class III); 43 patients (5.3%) with severe muscle weakness (Class IV) and for 25 patients a history of intubation (3.0%) (Class V) was documented. Disease severity at diagnosis was classified by assessment of QMG score and was available for 687 patients (84.4%) [22]. Median QMG score at diagnosis was 4 points (IQR 2.0–8.0).

With respect to ab status, 714 (87.6%) patients were seropositive, whereas 86 (10.5%) were seronegative. The ab-status included anti-AChR-ab (*n* = 641), anti-MuSK-ab (*n* = 71), and anti-LRP4-ab (*n* = 2). 436 patients (53.5%) received corticosteroids following diagnosis with a mean dosage of 15 mg (SD 10). The average time between diagnosis and the start of the first immunosuppressive therapy (IST) was 1.3 years (SD 3.7). 451 patients (54.6%) received their first IST less than 1 year after diagnosis and were considered as early IST, while 111 patients (13.5%) received IST after 1 year or more and were considered late IST. The remaining patients did not receive IST during the observation period.

### Predictive factors for MC and disease exacerbation

**Fig. 2:**
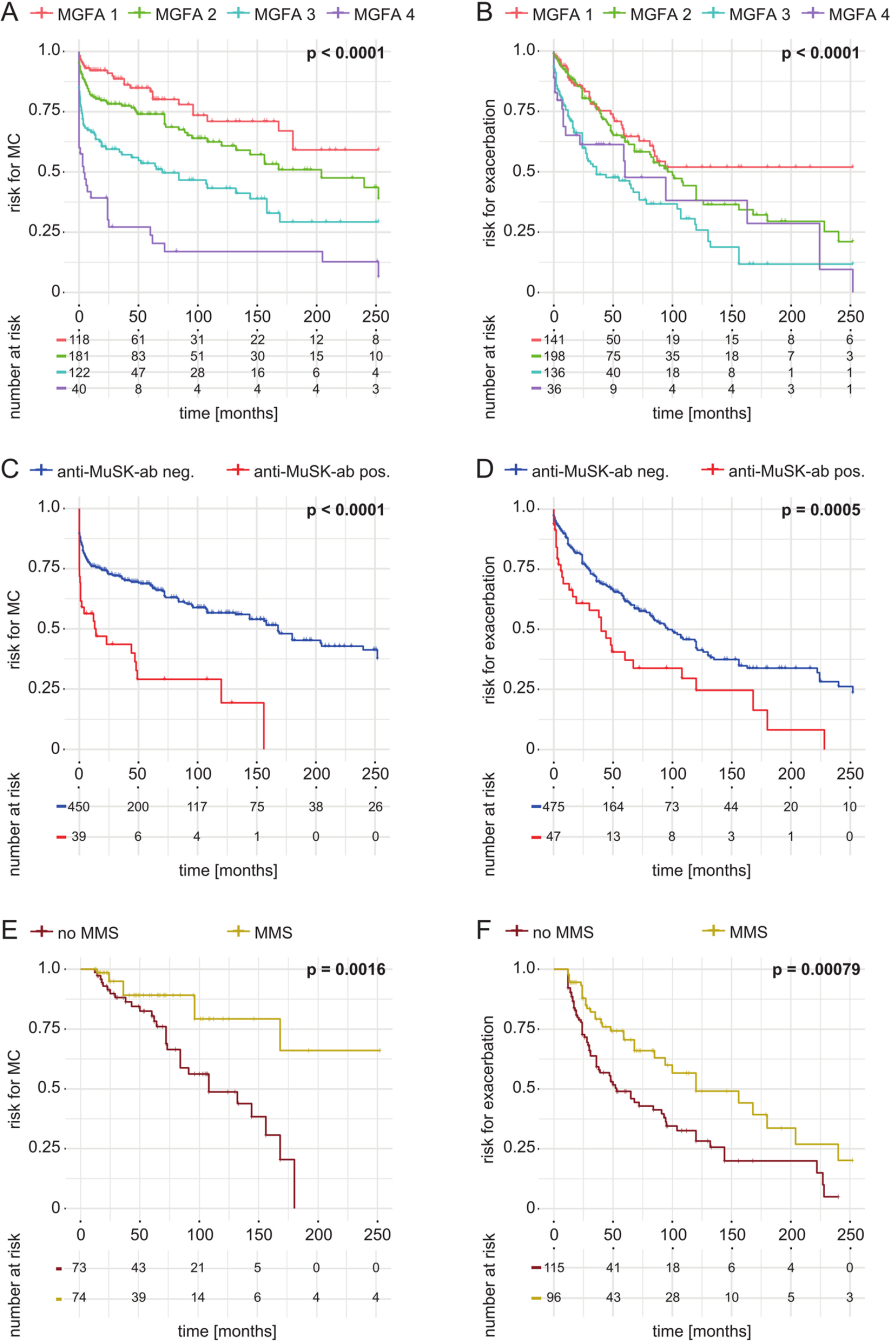
Survival analysis of MC and disease exacerbation. Survival curves displaying the time (in months) between diagnosis and the first MC (myasthenic crisis) or exacerbation. (**A**) Survival graph displaying the time to MC according to MGFA class. (**B**) Survival graph displaying the time to exacerbation according to MGFA class. (**C**) Survival graph displaying the time to MC according to anti-Musk-ab status. (**D**)Survival graph displaying the time to exacerbation according to anti-Musk-ab status. (**E**) Survival graph displaying the time to MC according to minimal manifestation status (MMS) at 12 months after diagnosis. (**F**) Survival graph displaying the time to exacerbation according to MMS at 12 months after diagnosis. Significance between survival curves was assessed by logrank testing. ****p < 0.0001 ***p < 0.001, **p < 0.01, *p < 0.05

Overall, 217 patients (26.3%) experienced a MC during their disease course while 225 patients (27.6%) experienced a disease exacerbation. To assess potential risk factors for the occurrence of MC or disease exacerbation, we employed a model of univariate logistic regression (Additional file 1: Table S1). We assessed the risk for experiencing at least one MC or disease exacerbation compared to patients experiencing no event. Aiming to identify independent risk factors, we entered risk factors reaching statistical significance (*p* < 0.05) in univariate analysis in a model of multivariate Cox regression. In addition, we included clinical parameters (sex and age) as they were related to clinical outcomes in previous studies [5]. To avoid overfitting, factors facing high collinearity were excluded (age at manifestation, thymectomy, imaging suggestive of thymoma). Accordingly, multivariate analysis revealed that QMG score at diagnosis [OR 1.23 95% confidence interval (95%CI) 1.14–1.66, *p* < 0.0001], MGFA class at diagnosis (OR 1.83 95% CI 1.65–1.97, *p* < 0.001), anti-MuSK-ab (OR 2.18 95% CI 1.76–2.59, *p* < 0.05) and the presence of a thymoma (OR 3.71 95% CI 3.01–4.41, *p* < 0.0001) predicted the occurrence of MC as independent risk factors (Table 2). Multivariate analysis of risk factors for disease exacerbation identified generalized disease (OR 1.83 95% CI 1.23–2.39, *p* < 0.05), QMG score at diagnosis (OR 1.12 95% CI 1.09 to 1.44, *p* < 0.001), anti-MuSK-ab (OR 1.07 95% CI 1.01–1.28, *p* < 0.01) and the presence of a thymoma (OR 1.56 95% CI 1.29–2.07, *p* < 0.05) as independent risk factors. Next, we applied the Kaplan–Meier method to our data set. Here, we observed an inverse relation between MGFA class and the occurrence of MC (Fig. 2A, B). In addition, we observed that anti-MuSK-ab status correlates with the risk for experiencing MC (Fig. 2C) or disease exacerbation (Fig. 2D) (Table 2).

**Table 2:** Risk factors for MC and exacerbation—Multivariate analysis

	Odds ratio	95%CI	*p*-value
MC			
Age at diagnosis	1.01	0.87–1.25	0.32
Sex	0.96	0.81–1.43	0.86
QMG score at diagnosis	1.23	1.14–1.66	**< 0.0001**
MGFA status at diagnosis	1.83	1.65–1.97	**< 0.0001**
Anti-MuSK-ab	2.18	1.76–2.59	**0.02**
Thymoma	3.71	3.01–4.41	**< 0.0001**
Cardiovascular disease	1.29	0.72–1.66	0.35
Heart failure (any cause)	1.11	0.71–1.78	0.48
Pulmonary disease	1.36	0.88–1.44	0.25
Chronic obstructive pulmonary disease	1.41	0.91–1.48	0.11
Exacerbation			
Sex	0.82	0.66–1.17	0.24
Age at diagnosis	1.03	0.76–1.51	0.45
Generalized disease at diagnosis	1.83	1.23–2.39	**0.03**
QMG score at diagnosis	1.12	1.09–1.44	**< 0.0001**
MGFA status at diagnosis	1.03	0.75–1.48	0.11
Anti-MuSK-ab	1.07	1.01–1.28	**0.003**
Thymoma	1.64	1.29–2.07	**0.02**
Pulmonary disease	1.22	0.71–1.47	0.32
Chronic obstructive pulmonary disease	1.32	0.92–1.41	0.12

Risk factors for MC and exacerbation in multivariate Cox regression analysis. *anti-Musk-ab* anti-muscle-specific tyrosine kinase-ab, *MGFA* Myasthenia Gravis Foundation of America, *SD* standard deviation, *QMG* quantitative myasthenia gravis score. Variables with a *p*-value < 0.05 in the univariate analysis and clinically relevant variables (sex, age) were included in the multivariate analysis. For highly collinear factors (thymoma, thymectomy and imaging suspect for thymoma as well as age at onset, age at diagnosis and early onset) we included only one variable to avoid overfitting. Risk is presented as odds ratio. A *p*-value below 0.05 was considered statistically significant. Statistically significant results are bold. 95% CI = 95% confidence interval

Finally, we investigated whether therapeutic management of MG influences the occurrence of MC and exacerbation during the disease course. To assess the time between diagnosis and treatment as a potential risk factor, we separated the patient cohort by the time between diagnosis and the start of the first standard IST. Standard IST comprised of azathioprine, MMF, methotrexate and cyclosporine. Here, the risk for MC and disease exacerbation was not different for patients with early vs. late IST, respectively (MC: OR 0.38 95% CI 0.22–0.87, *p* = 0.79, exacerbation: OR 0.86 95% CI 0.65–0.99, *p* = 0.38). In addition to the time to treatment, we investigated the effect of treatment response on the occurrence of MC or exacerbation. We analysed the risk for MC and exacerbation for patients achieving MMS at 12 months after diagnosis and those who did not. To exclude bias due to patients presenting with MC or exacerbation as first manifestation, patients with a clinical event up to 6 months after diagnosis were excluded from the analysis of treatment response. Indeed, the risk was reduced for achieving MMS for MC (OR 0.32 95% CI 0.17–0.61, *p* = 0.002) and for exacerbation (OR 0.50 95% CI 0.34–0.70, *p* < 0.001). Next, using the Kaplan–Meier method we observed treatment non-responders as at risk to experience MC and exacerbations early in their disease as compared to treatment responders (Fig. 2E, F). To further dissect the importance of therapeutic management, we analyzed both cortisone treatment, as binary variable, and dosage, as continuous variable, as predictors for MC or exacerbation. Here, the risk for MC (OR 1.12 95% CI 1.05–1.33, *p* = 0.16) and exacerbation (OR 1.09 95% CI 1.01–1.45, *p* = 0.42) were similar for patients receiving cortisone following diagnosis compared with those who did not. In the group of cortisone-treated patients, assessment of cortisone dose did not reveal an association with the risk for MC (OR 1.27 95% CI 1.16–1.65, *p* = 0.23) or exacerbation (OR 1.52 95% CI 1.34–1.72, *p* = 0.18).

### Factors determining the outcome of MC

Given the substantial mortality and lasting functional impairment associated with MC [5, 23], we further investigated potential factors affecting the outcome of MC. As detailed above, patients experiencing MC were grouped into three cohorts (improved, unchanged and worse). Clinical, demographic, diagnostic and therapeutic data were assessed for each cohort (Additional file 2: Table S2). Overall, 235 MC were recorded. In-hospital mortality was recorded for 6 patients (0.25%). To prevent bias (i.e., shorter ventilation time despite worse outcome), patients had died to MC were not included. Comparison of groups was performed on the remaining 229 MC. Recorded trigger factors are presented in Additional file 3: Table S3. Outcomes after MC were defined as improved for 143 MC (62.4%), unchanged for 33 MC (14.4%) and worse for 53 MC (23.2%) (Table 3).

**Table 3:** Factors affecting outcome of MC

	Improved (*p* value compared to worse)	Unchanged (*p* value compared to worse)	Worse
Female (% of patients)	51.5% (0.89^#^)	40.5% (0.62^#^)	48.1%
Age at MC [Year, mean (SD)]	57.9 (21.8) (**0.01**^**+**^)	60.7 (17.8) (0.14^+^)	67.5 (15.0)
Time between diagnosis and MC [Months, mean (SD)]	31.1 (50.7) (0.77^+^)	27.5 (61.0) (0.77^+^)	36.4 (52.5)
MGFA before MC [MGFA, median (IQR)]	2 (1) (**0.006**^**+**^)	2 (1) (0.38 +)	3 (2)
MGFA at admission [MGFA, median (IQR)]	3 (1) (**< 0.001**^**+**^)	3 (1) (**0.008**^**+**^)	4 (2)
Treated with IST at start of MD (% of patients)	63.1% (0.13^#^)	60.0% (0.49^#^)	50.9%
MC triggered by infection (% of MC triggered by infections)	33.5% (**0.005**^**#**^)	42.4% (0.26^#^)	56.6%
VC before MC [VC in ml, mean (SD)]	2192 (822) (0.14^+^)	1938 (739) (**< 0.001**^**+**^)	1533 (581)
VC at admission [VC in ml, mean (SD)]	1292 (800) (**0.04**^**+**^)	1263 (598) (**0.006**^**+**^)	871 (348)
Comorbidities [Number of comorbidities at admission, median (IQR)]	2 (2) (**< 0.001**^**+**^)	2 (2) (**< 0.001**^**+**^)	4 (2)
Time of hospitalisation [days, mean (SD)]	20.2 (16.1) (**< 0.001**^**+**^)	24.7 (25.8) (0.11^+^)	34.1 (32.4)
Intubated (% of patients)	26.9% (**< 0.001**^**#**^)	26.7% (**0.002**^**#**^)	64.2%
Time of invasive ventilation (days, mean)	7.8 (13.8) (**0.002**^**+**^)	4.1 (8.9) (**0.001**^**+**^)	22.6 (39.2)
Pneumonia (% of patients)	26.1% (**< 0.001**^**#**^)	20.0% (**< 0.001**^**#**^)	57.1%
Sepsis (% of patients)	6.6% (**0.002**^**#**^)	3.3% (**0.013**^**#**^)	25.0%
Treated with PLEX or IA (% of patients)	57.8% (0.11^#^)	45.5% (0.99^#^)	34.2%
Treated with IVIG and PLEX or IA (% of patients)	24.3% (0.24^#^)	12.2% (0.76^#^)	15.3%
Treated with no IVIG, PLEX or IA (% of patients)	18.0% (**< 0.001**^**#**^)	15.2% (**< 0.001**^**#**^)	60.3%

Factors affecting the outcome of MC. *SD* standard deviation, *IQR* interquartile range, *MC* myasthenic crisis, *MGFA* Myasthenia Gravis Foundation of America, *IST* immunosuppressive therapy, *IVIG* intravenous immunoglobulin, *IA* immunoadsorption, *PLEX* plasmapheresis, *VC* vital capacity. Patients who died during the MC were excluded from the analysis as to prevent bias of data due to early death. Significance for groups was assessed by ANOVA (denoted by ^+^) or Fisher’s exact test (denoted by ^#^). To account for multiple comparisons, statistical significance was corrected by the false discovery rate (FDR). A *p*-value below 0.05 was considered statistically significant. Statistically significant results are bold. Unless otherwise specified, values are mean ± SD (range), median ± IQR (range) or *n* (%)

Patients experiencing a worse outcome of following MC were older at the time of MC as compared to improved patients, while sex displayed no association with the outcome. MGFA class at admission as well as the last most recent measurement of MGFA class before prior to admission were lower in patients improving who improved. Interestingly, MC triggered by infections were was associated with a worse outcome. Consistent with previous reports, patients with a high number of comorbidities at admission had a worse outcome. Of note, vital capacity (VC) at admission, as well as the last recorded VC before MC, were was lower in patients worseningwho worsened. In addition, patients who were intubated, who had a longer time of mechanical ventilation or total hospital stay, and who developed pneumonia or sepsis had a poor outcome.

Finally, we analysed the impact of the available rescue therapies on the outcome of MC. Here, we compared the effect of IVIG (43 patients) vs. plasma exchange [PLEX (plasmapheresis) or IA (immunoadsorption)] (90 patients) vs. IVIG combined with plasma exchange (47 patients) vs. no rescue therapy (49 patients) (Table 3). Out of 49 patients with no rescue therapy, 31 were unable to receive therapy due to comorbidities (e.g., sepsis, renal failure), while 18 patient charts contained insufficient data on rescue treatments. Assessing the outcome of different rescue therapies revealed no differences between IVIG, plasma exchange, and the combination of both. However, patients receiving no rescue therapy had worse outcome compared to patients that received rescue therapy.

The six patients not surviving MC were on average 70 (SD 11.6) years. All 6 patients were intubated at admission, and the treating physician recorded an infection as the trigger for MC (pneumonia in all 6 cases). The average time of ventilation was 36.3 (SD32.5) days. Four patients died due to sepsis. One patient was treated with PLEX, one patient received both PLEX and IVIGs, while 4 patients did not receive rescue therapies.

## Discussion

Despite therapeutic advances, 10–20% of MG patients experience MC during their disease course [3, 6, 24]. To ameliorate the burden of disease incurred by uncontrolled disease, identification of patients at risk for these events as well as factors and strategies promoting MC remission are of high importance for clinical practice. To guide identification and—by extension—management of patients at risk, we analysed a large cohort of MG patients, which reflected previously reported demographic and clinical characteristics [6]. In essence, our data implicate disease severity at diagnosis as a readily accessible and reliable predictor for MC. Treatment strategies should be tailored to the severity of initial symptoms, potentially reducing the likelihood for MC or exacerbation. In addition, our data underlines that the prevention and resolution of infections are pivotal factors defining MC outcome.

Previous observational studies regarding possible risk factors are mostly available for the subgroup of MG patients that received thymectomy [11, 12]. Investigating patients with and without thymoma, our study corroborates the presence of thymoma as a risk factor. Corroborating previous studies [25, 26], we also identified anti-MuSK-ab positivity as an independent risk factor for disease deterioration. Anti-MuSK-ab positive has also been associated with poor outcome of MC [27]. Interestingly, disease severity as assessed by clinical scoring was a robust predictor for patients at risk for MC or exacerbation, underlining the importance of standardized clinical evaluation of MG patients. Patients presenting with severe disease should receive intensified disease monitoring to recognize and, if possible, prevent the occurrence of MC.

Analysing the impact of disease management, we observed that treatment response influenced the risk for MC. Here, patients achieving MMS were at an reduced risk for MC and exacerbation than those who did not. MMS was proposed by the International Consensus Guidance for Management of MG as treatment target [17, 20]. We analyzed this parameter to understand if achieving the proposed treatment target is associated with a reduced risk for MC [17]. Treatment strategies were previously suggested to affect the course of disease in MG. As such, a recent meta-analysis suggested that cortisone treatment reduces the risk for secondary generalization for MG patients with ocular manifestation [28]. Thymectomy is also evidenced to improve clinical readouts over a 3-year time span as demonstrated in a recent, randomized, controlled trial [19, 20]. Taken together, successful treatment approaches appear to influence long-term outcomes.

Knowledge of factors affecting the outcome of MC are of high clinical importance to promote remission and functional independence [6], with most studies reporting factors associated with prolonged ventilation as a surrogate marker for clinical outcome of MC [6, 23, 29]. Following analysis of patients experiencing MC according to the MGFA post-intervention-status [17], we observed an association between prolonged ventilation time and a worse outcome, suggesting that ventilation time correlates with functional status at discharge. However, our cohort also revealed that VC might be a valuable biomarker for risk stratification of MC as VC predicted the outcome if assessed at admission. Interestingly, a previous retrospective cohort analysing 5 patients with MC found no link between VC and the need for mechanical ventilation [30]. Corroborating VC as a predictive biomarker in other neuromuscular diseases such as Guillain-Barré syndrome [31], our study contrasts the findings from the previous cohort with the difference potentially attributed to the substantial variance in cohort size implicating that monitoring and improvement of ventilation might allow clinicians to avert severe courses of MC. Intriguingly, an infectious trigger of MC was both frequent and associated with an unfavourable outcome compared to other triggers. Hence, prevention and early management of infection in MG patients, notably in MG patients with impaired ventilatory capacities, constitutes a cornerstone in the management of MC. We suggest that treatment of comorbidities making patients vulnerable to infection and resolute adhesion to vaccination protocols should be employed to reduce the risk of infection for MG patients.

The retrospective design of this study might be vulnerable to confounding factors as data were collected during routine clinical practice rather than a formal study setting making data sensitive to variation both in quantity and quality between individual patients and time points. Nonetheless, data quality was improved by collection according to standardised requirements of the German Myasthenia register. A focus on tertiary centers might introduce a bias towards severe cases. However, given the rarity of the disorder, most MG patients are treated in specialized centres [32]. Thus, our cohort is likely to be representative of the general MG population. Regarding the analysis of predictors, a potential limitation is that patients initially presenting with MC or exacerbation could not be included. These patients potentially constitute a distinct clinical subtype as they are expected to have fewer co-morbidities and are likely to be treated more aggressively [6]. Furthermore, definitions for MG exacerbation are heterogenous and diverging interpretations have been previously proposed, e.g., de Meel et al. included an increase in immunosuppressive therapy in their operational definition for exacerbation [33]. A caveat to the analysis of rescue therapies is that the subgroup of patients receiving no treatment for MC is biased to severe cases as these patients were often unable to be treated due to comorbidities (e.g., sepsis or renal failure).

## Conclusions

Our study highlights that disease severity at diagnosis is a valuable clinical marker to identify patients at risk for MC or disease exacerbation. Intensified monitoring with emphasis on the prevention of infectious complications is pivotal for management of patients at risk.

## Supplementary Information

**Additional file 1.** **Suppl. Table 1.** Risk factors for MC and exacerbation – Univariate analysis.

**Additional file 2.** **Suppl. Table 2.** Clinical and demographic characteristics of included MC.

**Additional file 3.** **Suppl. Table 3.** Trigger factors.

## Data Availability

The datasets used and/or analysed during the current study are available from the corresponding author on reasonable request.

## Abbreviations

ANOVA: Analysis of variance
ab: Antibody
anti-AChR-ab: Anti-acetylcholine-receptor-ab
anti-LRP4-ab: Anti-low-density lipoprotein receptor-related protein 4-ab
anti-MuSK-ab: Anti-muscle-specific tyrosine kinase-ab
anti-VGCC-ab: Anti-voltage-gated calcium channel-ab
EOMG: Early-onset MG
FDR: False discovery rate
IA: Immunoadsorption
iMC: Integrated Myasthenia Centre
IQR: Interquartile range
IST: Immunosuppressive therapy
IVIG: Intravenous immunoglobulin
LOMG: Late-onset MG
MC: Myasthenic crisis
MG: Myasthenia gravis
MGFA: Myasthenia Gravis Foundation of America
MGFA–PIS: Myasthenia Gravis Foundation of America post-intervention status
MMF: Mycophenolate-mofetil
NICU: Neurological Intensive Care Unit
OR: Odds ratio
PLEX: Plasmapheresis
SD: Standard deviation
