# Supplementary material for: Independent risk factors for myasthenic crisis and disease exacerbation in a retrospective cohort of myasthenia gravis patients
Source: J Neuroinflammation. 2022 Apr 12;19:89. doi: 10.1186/s12974-022-02448-4 (PMC9005160; doi:10.1186/s12974-022-02448-4)
Supplement: Supplementary file 1 — Additional file 1. Suppl. Table 1. Risk factors for MC and exacerbation – Univariate analysis. [file 12974_2022_2448_MOESM1_ESM.docx]

| Suppl. Table 1 Risk factors for MC and exacerbation – Univariate analysis | | | | | | |
| --- | --- | --- | --- | --- | --- | --- |
|  | **MC** | | | **Exacerbation** | | |
|  | **Odds ratio** | **95%CI** | **p-value** | **Odds ratio** | **95%CI** | **p-value** |
| Baseline data | | | | | | |
| Male | 1.21 | 0.88 to 1.65 | 0.23 | 0.62 | 0.48 to 1.11 | 0.09 |
| Age at manifestation  (years) | 1.02 | 1.01 to 1.03 | **<0.0001** | 0.99 | 0.98 to 1.00 | 0.06 |
| Age at diagnosis  (years) | 1.02 | 1.01 to 1.03 | **<0.0001** | 0.99 | 0.98 to 1.00 | 0.06 |
| Early onset | 0.53 | 0.37 to 0.75 | **0.0003** | 0.97 | 0.96 to 1.00 | 0.08 |
| Disease severity at diagnosis | | | | | | |
| Generalized disease at diagnosis | 1.51 | 0.97 to 3.00 | 0.06 | 2.75 | 1.79 to 4.34 | **<0.0001** |
| QMG score at diagnosis | 1.28 | 1.23 to 1.35 | **<0.0001** | 1.11 | 1.09 to 1.16 | **<0.0001** |
| MGFA status at diagnosis | 2.89 | 2.39 to 3.52 | **<0.0001** | 1.47 | 1.29 to 1.73 | **<0.0001** |
| Serological status | | | | | | |
| Seropositive | 1.18 | 0.79 to 1.76 | 0.41 | 0.6 | 0.53 to 1.15 | 0.09 |
| Anti-AChR-ab | 1.45 | 0.99 to 2.11 | 0.06 | 1.3 | 0.84 to 1.31 | 0.73 |
| Anti-MuSK-ab | 1.87 | 1.11 to 3.08 | **0.02** | 1.97 | 1.20 to 3.23 | **0.007** |
| Anti-Titin-ab | 1.39 | 0.98 to 2.15 | 0.07 | 1.1 | 0.97 to 1.11 | 0.83 |
| Diagnostic findings | | | | | | |
| Incremental response | 1.31 | 0.38 to 4.44 | 0.61 | 1.17 | 0.98 to 1.11 | 0.74 |
| Decremental response | 1.89 | 0.97 to 4.50 | 0.08 | 1.12 | 0.75 to 1.33 | 0.84 |
| Positive edrophonium test | 0.89 | 0.41 to 1.12 | 0.26 | 0.75 | 0.58 to 1.10 | 0.51 |
| Imaging suspect for thymoma | 1.68 | 1.24 to 2.29 | **0.0009** | 1.41 | 1.01 to 1.88 | 0.019 |
| Thymoma status | | | | | | |
| Thymoma | 1.94 | 1.34 to 2.81 | **0.0004** | 1.99 | 1.35 to 2.86 | **0.0005** |
| Thymectomy | 1.6 | 1.04 to 1.97 | **0.03** | 1.64 | 1.21 to 2.32 | **0.001** |
| Comorbidities | | | | | | |
| Autoimmune disease | 1.20 | 0.88 to 1.74 | 0.31 | 0.65 | 0.39 to 1.11 | 0.19 |
| Cardiovascular disease | 2.45 | 1.82 to 3.26 | **<0.0001** | 1.23 | 0.99 to 1.85 | 0.06 |
| Pulmonary disease | 2.19 | 1.49 to 3.21 | **<0.0001** | 1.50 | 1.04 to 2.21 | **0.04** |
| Malignancy other than thymoma | 1.22 | 0.70 to 2.50 | 0.49 | 1.61 | 0.78 to 2.11 | 0.44 |
| Metabolic disease | 1.36 | 0.98 to 3.71 | 0.13 | 1.61 | 0.87 to 2.23 | 0.11 |
| Gastrointestinal disease | 1.76 | 0.91 to 2.13 | 0.06 | 1.11 | 0.91 to 1.87 | 0.15 |

**Risk factors for MC and exacerbation in univariate analysis.** Abbreviations: anti-AChR-ab = anti-acetylcholine-receptor-ab; anti-Musk-ab = anti-muscle-specific tyrosine kinase-ab; anti-LRP4-ab = anti-low-density lipoprotein receptor-related protein 4-ab; 95%CI = 95% confidence interval; MC = myasthenic crisis. The likelihood ratio test was used for statistical analysis of possible risk factors in univariate logistic regression. Risk is presented as Odds ratio. A p-value below 0.05 was considered statistically significant. Statistically significant results are bold.
