# Supplementary material for: Independent risk factors for myasthenic crisis and disease exacerbation in a retrospective cohort of myasthenia gravis patients
Source: J Neuroinflammation. 2022 Apr 12;19:89. doi: 10.1186/s12974-022-02448-4 (PMC9005160; doi:10.1186/s12974-022-02448-4)
Supplement: Supplementary file 2 — Additional file 2. Suppl. Table 2. Clinical and demographic characteristics of included MC. [file 12974_2022_2448_MOESM2_ESM.docx]

| **Suppl. Table 2** | | |
| --- | --- | --- |
|  | **n** | **%** |
| Total number of MC | 235 | 100 |
| Experienced a previous MC | 25 | 10.6 |
| Male/Female | 113/122 | 48.1/51.9 |
| MC as first manifestation | 21 | 8.9 |
| Duration of hospitalisation, days (mean) | 24.5 (23.1) |  |
| Duration of ventilation, days (mean) | 11.9 (24.9) |  |
| In-hospital mortality | 8 | 3.4 |
| Time between diagnosis and first MC, months (mean) | 34.0 (53.5) |  |
| Age at first MC, years (mean) | 60.8 (20.1) |  |
| Vital capacity at start of MC, ml (mean) | 1043 (721) |  |
| Experienced pneumonia | 74 | 31.5 |
| Experienced sepsis | 28 | 11.9 |
| Number of comorbidities, median (IQR) | 2 (4) |  |
| Treatment of crisis | | |
| IA or PLEX | 90 | 38.3 |
| IVIG | 43 | 18.3 |
| IVIG and IA or PLEX | 47 | 20.0 |
| No rescue treatment | 49 | 20.8 |
| Outcome | | |
| Improved | 143 | 60.8 |
| Unchanged | 33 | 14.0 |
| Worsened | 53 | 22.5 |
| Died | 6 | 2.5 |
| **Clinical and demographic characteristics of included MC.** Abbreviations: MC = myasthenic crisis; MG = myasthenia gravis; IVIG = intravenous immunoglobulin; IA = immunoadsorption; IQR = interquartile range; PLEX = plasmapheresis; SD = standard deviation. Unless otherwise specified, values are mean ± SD, median ± IQR or n (%). | | |
