# Supplementary material for: Independent risk factors for myasthenic crisis and disease exacerbation in a retrospective cohort of myasthenia gravis patients
Source: J Neuroinflammation. 2022 Apr 12;19:89. doi: 10.1186/s12974-022-02448-4 (PMC9005160; doi:10.1186/s12974-022-02448-4)
Supplement: Supplementary file 3 — Additional file 3. Suppl. Table 3. Trigger factors. [file 12974_2022_2448_MOESM3_ESM.docx]

| **Suppl. Table 3 Trigger factors** | | |
| --- | --- | --- |
|  | **Precipitating factors of MC (%)** | **Precipitating factors of disease exacerbation (%)** |
| Infection | 98 (41.7) | 93 (21.5) |
| Insufficient immunosuppression | 17 (7.2) | 163 (37.6) |
| Incompliance | 9 (3.8) | 18 (4.0) |
| Comorbidity | 9 (3.8) | 45 (10.3) |
| Post-surgery | 17 (7.2) | 25 (5.0) |
| Unknown | 85 (36.2) | 89 (20.5) |
| **Events in total** | **235 (100)** | **433 (100)** |

**Trigger factors of MC and disease exacerbations as recorded by the treating physician.** Abbreviations: MC = myasthenic crisis; Values are n (%).
